# Neuroprotective Effects of HSF1 in Retinal Ischemia-Reperfusion Injury

**DOI:** 10.1167/iovs.18-26216

**Published:** 2019-03

**Authors:** Wei Liu, Fan Xia, Yonju Ha, Shuang Zhu, Yi Li, Oluwarotimi Folorunso, Aryan Pashaei-Marandi, Pei-Yi Lin, Ronald G. Tilton, Anson P. Pierce, Hua Liu, Wenbo Zhang

**Affiliations:** 1 Department of Ophthalmology & Visual Sciences, University of Texas Medical Branch, Galveston, Texas, United States; 2 Department of Ophthalmology, Union Hospital, Tongji Medical College, Huazhong University of Science and Technology, Wuhan, China; 3 Eye Hospital, Tianjin Medical University, Tianjin, China; 4 Department of Pharmacology and Toxicology, University of Texas Medical Branch, Galveston, Texas, United States; 5 Thermo Fisher Scientific, Grand Island, New York, United States; 6 Internal Medicine, University of Texas Medical Branch, Galveston, Texas, United States; 7 Departments of Neuroscience, Cell Biology & Anatomy, University of Texas Medical Branch, Galveston, Texas, United States

**Keywords:** retinal ischemia, HSF1, neuronal injury, heat shock protein; ER stress

## Abstract

**Purpose:**

Retinal ischemia, a common cause of several vision-threatening diseases, contributes to the death of retinal neurons, particularly retinal ganglion cells (RGCs). Heat shock transcription factor 1 (HSF1), a stress-responsive protein, has been shown to be important in response to cellular stress stimuli, including ischemia. This study is to investigate whether HSF1 has a role in retinal neuronal injury in a mouse model of retinal ischemia-reperfusion (IR).

**Methods:**

IR was induced by inserting an infusion needle into the anterior chamber of the right eye and elevating a saline reservoir connected to the needle to raise the intraocular pressure to 110 mm Hg for 45 minutes. HSF1, Hsp70, molecules in the endoplasmic reticulum (ER) stress branches, tau phosphorylation, inflammatory molecules, and RGC injury were determined by immunohistochemistry, Western blot, or quantitative PCR.

**Results:**

HSF1 expression was significantly increased in the retina 6 hours after IR. Using our novel transgenic mice carrying full-length human HSF gene, we demonstrated that IR-induced retinal neuronal apoptosis and necroptosis were abrogated 12 hours after IR. RGCs and their function were preserved in the HSF1 transgenic mice 7 days after IR. Mechanistically, the beneficial effects of HSF1 may be mediated by its induction of chaperone protein Hsp70 and alleviation of ER stress, leading to decreased tau phosphorylation and attenuated inflammatory response 12 to 24 hours after IR.

**Conclusions:**

These data provide compelling evidence that HSF1 is neuroprotective against retinal IR injury, and boosting HSF1 expression may be a beneficial strategy to limit neuronal degeneration in retinal diseases.

Retinal ischemia occurs when retinal blood supply is impaired by retinal vascular occlusion, injury or regression, or by increased intraocular pressure. It is a common cause of visual impairment and blindness in various forms of ischemic retinopathy including central retinal artery occlusion, retinal vein occlusion, glaucoma, traumatic optic neuropathy (TON), and diabetic retinopathy.^1^,^2^ Retinal neurons, particularly retinal ganglion cells (RGCs), are vulnerable to ischemia and all of these diseases lead to thinning of the nerve fiber layer as a result of ganglion cell injury or loss.^3^,^4^ To date, there are no clinically approved drugs that can effectively save retinal neurons in ischemic retinopathy. A better understanding of the molecular mechanisms regulating retinal injury after retinal ischemia is important for the development of new therapies to treat, minimize and/or reverse the degree of vision loss due to retinal neuronal injury.

Heat shock transcription factor 1 (HSF1) is a master transcriptional regulator that modulates the expression of chaperones in response to stresses such as elevated temperature, oxidative stress, and exposure to proteotoxic agents.^5^–^7^ HSF1 is maintained in cytoplasm as an inactive monomer by interaction with a multi-chaperone complex composed of heat shock proteins (HSPs) 40, 70, and 90, and the cytosolic chaperonin TCP1 ring complex (TRiC). Upon cell stress, misfolded proteins are generated and bind to HSPs and TRiC, which frees monomeric HSF1 from repression by chaperones, leading to HSF1 trimerization and activation. Subsequently, HSF1 is translocated to the nucleus where it binds to conserved heat shock elements and drives transcription of target genes including HSPs. HSPs are essential for protein quality control by assisting protein folding or directing misfolded proteins to appropriate degradative pathways, including the proteasome and or lysosome.^6^ Through induction of HSPs, HSF1 plays a critical role in enhancing cell survival under stressful conditions. Consequently, the impairment of HSF1 activity has been linked to neuronal dysfunction and cell death in a variety of neurodegenerative diseases such as Huntington's, Parkinson's, and Alzheimer's disease. In contrast, overactive HSF1 is involved in cancer by promoting cancer cell adaptation and survival in stressful conditions.^5^ It has been found that HSPs, including Hsp70, α-crystallins, Hsp40, and Hsp110, were able to enhance the survival of retinal neurons in models of glaucoma,^8^,^9^ optic nerve crush,^9^ polyglutamine disease,^9^,^10^ autoimmune uveitis,^11^,^12^ and retinal detachment.^13^ Moreover, inhibition of Hsp90 has neuroprotective effects in inherited retinal degeneration and polyglutamine diseases at least partially mediated by HSF1.^14^,^15^ However, a direct assessment of HSF1 in retinal diseases has never been performed and the role of HSF1 in ischemic retinopathy is unknown.

In this study, we found that HSF1 is transiently upregulated after retinal ischemic injury. Using a transgenic mouse carrying full-length human HSF gene, we demonstrated that boosting HSF1 expression induces Hsp70 expression, prevents endoplasmic reticulum (ER) stress, abrogates tau phosphorylation and inflammation, and ultimately promotes neuronal survival after retinal ischemia.

## Methods

### Animals

Animal protocols were approved by the Institutional Animal Care and Use Committee of the University of Texas Medical Branch. All experimental procedures and use of animals were performed in accordance with the Association for Research in Vision and Ophthalmology Statement for the Use of Animals in Ophthalmic and Vision Research. C57BL/6J mice were purchased from Jackson Laboratory (Bar Harbor, ME, USA) and HSF1 transgenic mice (HSF1-Tg) mice were generated as described previously.^6^ HSF1-Tg mice were crossed with C57BL/6J mice to get both WT and Tg littermates for experiments. Mice were maintained on a 12:12 light/dark cycle with food and water available ad libitum.

### Induction of Ischemia-Reperfusion (IR)

IR was induced as previously described.^16^ Briefly, 8- to 12-week-old mice were anesthetized with a mixture of 100 mg/kg ketamine hydrochloride and 10 mg/kg xylazine hydrochloride by intraperitoneal (IP) injection. Then 0.5% proparacaine hydrochloride was applied topically. The anterior chamber of the right eye was cannulated with a 30-gauge infusion needle connected to a saline reservoir. The intraocular pressure was raised to 110 mm Hg by elevating the saline reservoir for 45 minutes. The left eye without elevating the pressure served as control. Mice were kept on a circulating water warming pad to maintain a constant body temperature of 37.0°C. Eyeballs or retinas were collected at the indicated time points after IR for further analysis.

### Induction of Traumatic Optic Neuropathy

Optic nerve crush (ONC) is an established method for generating the model of TON.^17^,^18^ Mice (8- to 12-week-olds) were anesthetized by intraperitoneal injection of a mixture of 100 mg/kg ketamine hydrochloride and 10 mg/kg xylazine hydrochloride. For local anesthesia, 0.5% proparacaine was applied to the eye before the procedure. After a small incision was made by cutting conjunctiva around the eye globe, the right optic nerve close to its origin in the optic disk was crushed for 10 seconds using forceps (Dumont RS5005; Roboz, Gaithersburg, MD, USA). The left eye without crushing served as control. Eyeballs were collected at 7 days after ONC procedure for further analysis.

### High Resolution En Face Optical Coherence Tomography (OCT)

Seven days after IR, mice were anesthetized with an intraperitoneal injection of a mixture of 100 mg/kg ketamine and 10 mg/kg xylazine. Pupils were dilated with tropicamide and phenylephrine and high resolution OCT analysis was performed with a spectral domain ophthalmic imaging system (Envisu R2200; Bioptigen, Inc., Morrisville, NC, USA) as described previously.^18^,^19^ Retinal thickness maps and statistics were obtained using Bioptigen's report generator. The ganglion cell complex (GCC) is defined as the three innermost retinal layers: the nerve fiber layer, the ganglion cell layer and the inner plexiform layer.

### Immunostaining of Retinal Whole Mounts

Eyeballs were collected and fixed in 4% paraformaldehyde (PFA) at 4°C overnight. Next day, retinas were dissected, washed with PBS, blocked and permeabilized with PBS containing 5% normal goat serum and 0.3% Triton X-100 for 3 hours. Subsequently, retinas were incubated with primary antibody against Tuj1 (1:400, BioLegend, San Diego, CA, USA) or CD45 (1:400, BD Biosciences, San Jose, CA, USA) at 4°C overnight. After washing, retinas were then incubated with appropriate AlexaFluor 488 or 594-conjugated secondary antibodies (1:400, Thermo Fisher Scientific, Waltham, MA, USA) at 4°C for 4 hours. Finally, retinas were mounted and images were captured with confocal microscopy (LSM 510 Meta, Carl Zeiss, Inc., Thornwood, NY, USA).

### Analysis of Leukostasis

Retinal leukostasis was assayed by labeling the leukocytes adherent to retinal endothelium.^20^ At 24 hours after IR procedure, mice were deeply anesthetized by intraperitoneal injection of a mixture of 100 mg/kg ketamine hydrochloride and 10 mg/kg xylazine hydrochloride and the chest cavity was carefully opened. The perfusion cannula was inserted into the aorta and drainage was achieved by opening the right atrium. PBS was perfused to wash out nonadherent blood cells. Then, FITC-labeled concanavalin A (Con A) lectin (40 μg/mL in PBS, pH 7.4; Vector Laboratories, Burlingame, CA, USA) was perfused to label the adherent leukocytes and vasculature. Next, PBS was perfused to remove the residual unbound Con A. Finally, eyeballs were removed and fixed with 4% PFA. Retinas were dissected and stained with anti-CD45 antibody. The total number of the adherent leukocytes per retina and leukocytes infiltrated into the retina were counted under fluorescence microscopy.

### Immunostaining of Retinal Frozen Section

Following fixation with 4% PFA in 0.1 M phosphate buffer for 60 minutes, eyeballs were equilibrated in 30% sucrose overnight, and embedded in optimal cutting temperature compound (OCT). Cryosections (10 μm) were obtained, postfixed with 4% PFA for 10 minutes, rinsed with PBS, permeabilized with PBS containing 0.1% Triton X-100 for 15 minutes at room temperature, and blocked with PowerBlock (BiogenX, San Ramon, CA, USA) for 1 hour. Next, sections were probed with the following primary antibodies: HSF1 (Enzo Life Sciences, Farmingdale, NY, USA), Hsp70 (Enzo Life Sciences), Grp78 (BD Biosciences), Chop (Santa Cruz Biotechnology, Dallas, TX, USA), p-Perk (Cell Signaling Technology, Beverly, MA, USA) and AT180 (Thermo Fisher Scientific). After washing with PBS, sections were incubated with appropriate AlexaFluor 488-conjugated secondary antibodies (Thermo Fisher Scientific), mounted with medium containing DAPI (Abcam, Cambridge, MA, USA) or nuclei were counterstained with propidium iodide (PI; Fisher Scientific International, Inc., Hampton, NH, USA). Images were taken with epifluorescence microscopy.

### TUNEL Assay

TUNEL staining was performed on retinal frozen sections with a commercial detection kit (ApopTag Fluorescein In Situ Apoptosis Detection Kit; EMD Millipore, Billerica, MA, USA) according to the manufacturer's instructions. Retinal sections were counterstained with PI to label nuclei and TUNEL-positive cells were counted under epifluorescence microscope.

### Propidium Iodide Uptake

PI uptake assay was performed to label necrotic cells.^2^,^21^ Briefly, mice were subjected to IR injury and 5 mg/kg PI was injected intraperitoneally at 9 hours after the IR. Three hours later, eyeballs were harvested, embedded in OCT compound, and cryosectioned. The total number of the PI-positive cells per section was counted under fluorescence microscopy.

### Western Blot Analysis

Retinal proteins were extracted in RIPA buffer (50 mM Tris-HCl pH 7.4, 150 mM NaCl, 0.25% deoxycholic acid, 1% NP-40, 1 mM EDTA) containing 1X protease and phosphatase inhibitors (Roche Applied Science, Indianapolis, IN, USA). Protein concentrations were determined with Pierce BCA Protein Assay Kit (Pierce, Rockford, IL, USA) and 10 μg proteins were run on 10% SDS-polyacrylamide gels. The primary antibodies included the following: HSF1 (Enzo Life Sciences) and AT8 (Thermo Fisher Scientific). Proteins were detected by enhanced chemiluminescence (Pierce Biotechnology) using either X-ray film or an imaging system (ChemiDoc XRS+; Bio-Rad Laboratories, Hercules, CA, USA) and quantified using ImageJ (http://imagej.nih.gov/ij/; provided in the public domain by the National Institutes of Health, Bethesda, MD, USA). To normalize for protein loading, chemiluminescence of the bands in each lane was standardized to the intensity of the β-actin (Sigma-Aldrich, St. Louis, MO, USA) band in the same lane. Protein expression was presented as relative to that of the control.

### Real Time Quantitative RT-PCR

Total retinal mRNA was isolated using a commercial kit (RNAqueous-4PCR; Thermo Fisher Scientific), quantified using a spectrophotometer (NanoDrop; Thermo Fisher Scientific), and converted to cDNA using High-Capacity cDNA Reverse Transcription Kit (Thermo Fisher Scientific). Quantitative PCR was performed with SYBR Green Master Mix (Applied Biosystems, Waltham, MA, USA) using a PCR system (StepOnePlus; Applied Biosystems). Primer sequences for mouse transcripts were as follows: Hprt For-5′-GAA AGA CTT GCT CGA GAT GTC ATG-3′; Hprt Rev-5′-CAC ACA GAG GGC CAC AAT GT-3′; Cxcl10 For-5′-CAT CCC TGC GAG CCT ATC C-3′; Cxcl10 Rev-5′-CAT CTC TGC TCA TCA TTC TTT TTC A-3′; iNos For-5′-GGC AGC CTG TGA GAC CTT TG-3′; iNos Rev-5′-TGC ATT GGA AGT GAA GCG TTT-3′; Il-1β For-5′-AGT TGA CGG ACC CCA AAA GA-3′; Il-1β Rev-5′-GGA CAG CCC AGG TCA AAG G-3′; HSF1 For-5′-TCG TGC GGC AGC TCA AC-3′; HSF1 Rev-5′-CAG GCC ACC CTG CTC AAT-3′; Hsp70 For-5′-GGC TGG TGA GCC ACT TCG T-3′; Hsp70 Rev-5′- GTT CTG GCT GAT GTC CTT CTT GT-3′. Primer sequences for human transcripts were as follows: HSF1 For-5′-CGA CAG TGG CTC AGC ACA TT-3′; HSF1 Rev-5′-GGA CGT GCT CCA GGG AGA A-3′. Primer sequences for both mouse/human transcripts were as follows HSF1 For-5′-AAG CTC ATT CAG TTC CTG ATC TCA-3′; HSF1 Rev-5′-CTT TCT CTT CAC CCC CAG GAT-3′. Data were normalized to the internal control Hprt and the fold difference in different transcripts was calculated by the ΔΔCT method.

### Dark-Adapted ERG Analysis

Mice were induced IR at 8 to 12 weeks of age and ERG analysis was performed during daytime at 7 days after IR as described previously.^17^,^18^ Briefly, mice were dark-adapted overnight, anesthetized by intraperitoneal injection of a mixture of 100 mg/kg ketamine hydrochloride and 10 mg/kg xylazine hydrochloride. Following pupil dilation with a mixture of atropine and phenylephrine, gold ring electrodes were placed on the center of the cornea and ERG were recorded using an electrophysiology system (Espion; Diagnosys LLC, Lowell, MA, USA). Mice were kept on a lab cradle with a self-heating platform to maintain a constant body temperature of 37.0°C. Brief white flashes were presented from dim to bright with the interstimulus interval. Positive scotopic threshold responses (pSTRs) were recorded in response to briefly flashed stimuli between −4.3 to −3.2 log cd-s/m^2^. pSTRs were measured from baseline to the positive peak of waveform at 110 ms after the flash onset. Each record was an average of at least 50 responses.

### Statistical Analysis

Statistical analysis was conducted using GraphPad Prism program (GraphPad Software, Inc., La Jolla, CA, USA). Results were presented as mean ± standard error of mean (SEM) and analyzed by Student's *t*-test or 1-way ANOVA followed by Newman Keuls post hoc test. A *P* value < 0.05 was considered statistically significant.

## Results

### HSF1 is Upregulated After Retinal Ischemia-Reperfusion

**Figure 1:**
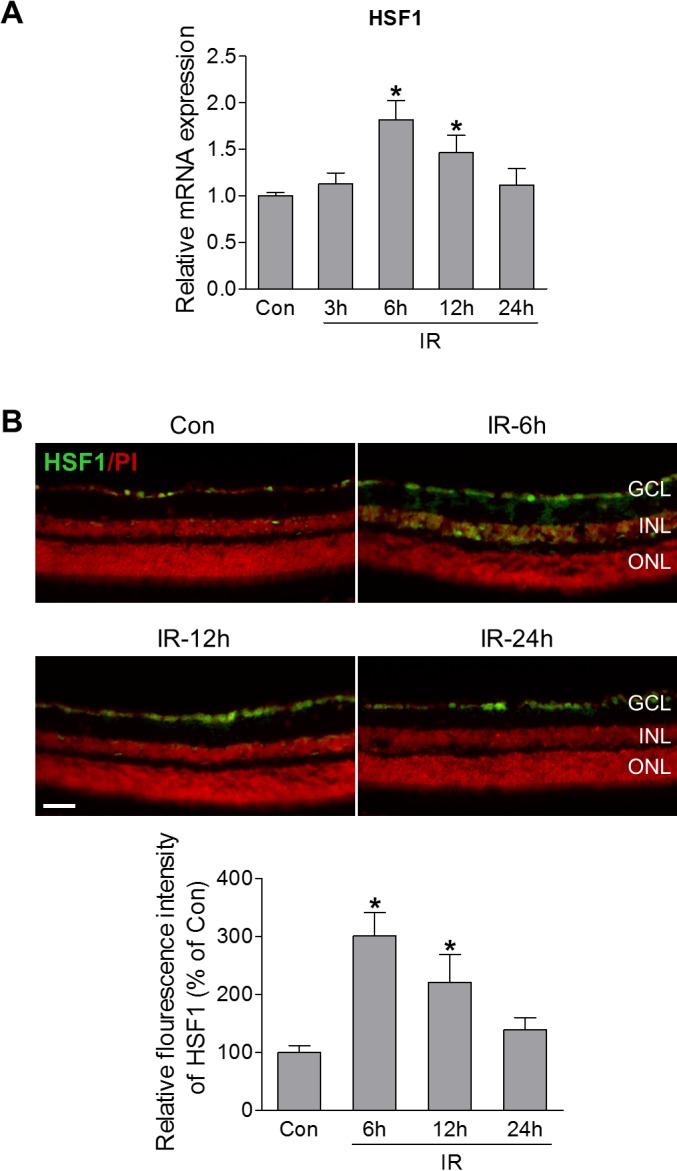
HSF1 expression is increased following retinal ischemia-reperfusion injury. WT mice were subjected to IR, and retinas or eyeballs were collected at various times after IR. (A) Quantitative PCR (qPCR) analysis of HSF1 mRNA expression in noninjured control retinas (Con) or injured retinas at 3, 6, 12, and 24 hours after IR. (B) Representative images of HSF1 immunostaining (green) in retinal frozen sections from control and injured-eyes at 6, 12, and 24 hours after IR. Red is PI staining. Bar graph represents quantification of immunoreactivity of HSF1 protein. N = 3 to 4 mice; *P < 0.05 versus control. Scale bar: 50 μm. ONL, outer nuclear layer.

To investigate whether HSF1 is implicated in retinal ischemia, we used a mouse model of ischemia-reperfusion (IR), in which retinal ischemia is induced by acute elevation of intraocular pressure,^22^ and examined the expression of HSF1. As shown in Figure 1A, transient ischemia induced an upregulation of HSF1 mRNA in the retinas at 6 hours and 12 hours after IR injury, followed by a return to normal level at 24 hours after injury. Consistent with the upregulation of mRNA, HSF1 immunoreactivity was significantly increased in neurons of the ganglion cell layer (GCL) and moderately increased in the cells of the inner nuclear layer (INL) at 6 and 12 hours after injury as determined by immunostaining (Fig. 1B). HSF1-positive cells include RGCs, amacrine cells, and bipolar cells (Supplementary Fig. S1). These data indicate that HSF1 expression changes in response to ischemic injury, and suggest a potential role in ischemic retinopathy.

### HSF1 Overexpression Prevents RGC Loss and Dysfunction After IR

**Figure 2:**
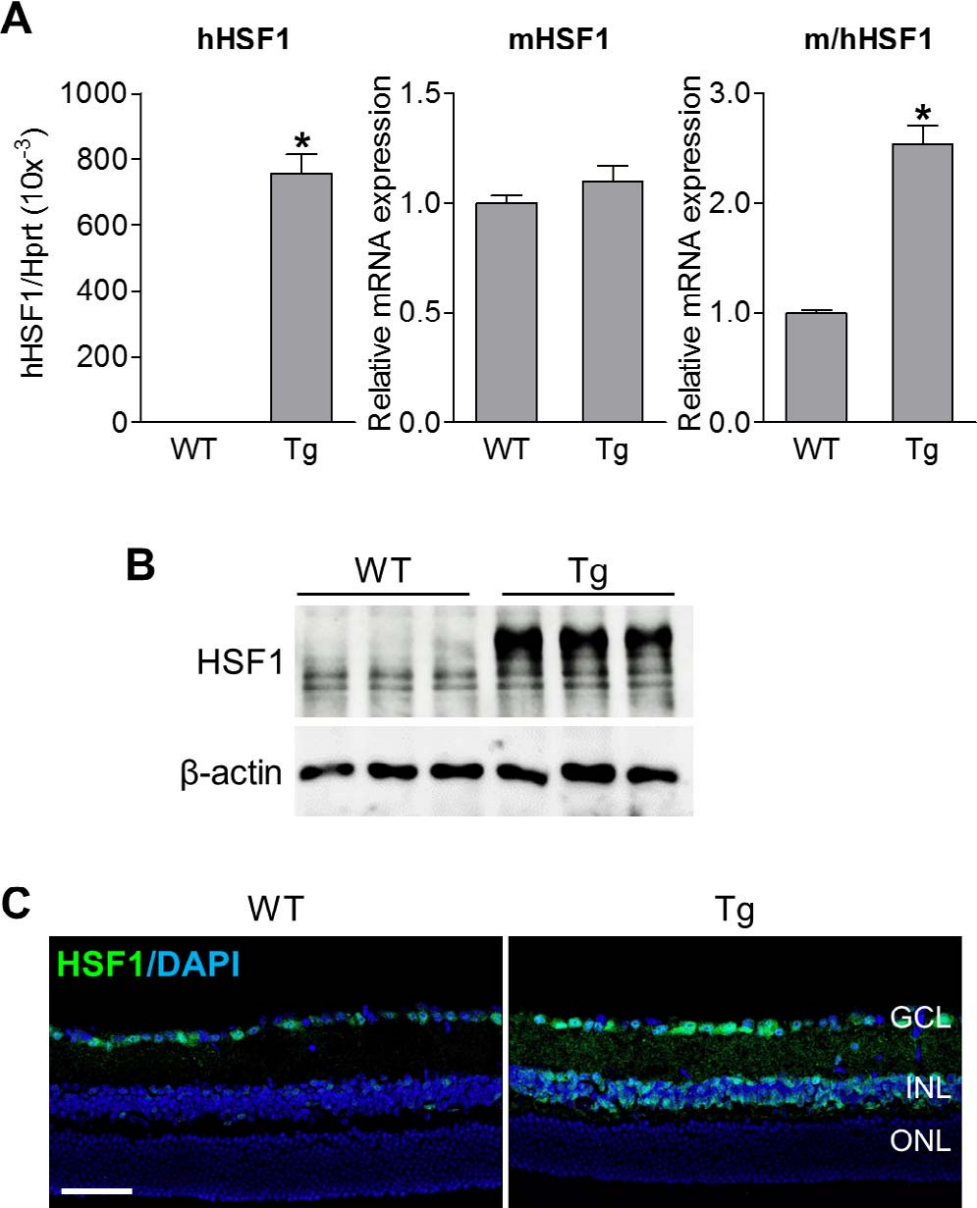
Confirmation of HSF1 overexpression in the retina of HSF1-Tg mice. Retinas or eyeballs were collected from WT and HSF-Tg mice. (A) Quantitative PCR analysis for mRNA expression of human HSF1 (hHSF1), murine HSF1 (mHSF1) or both (m/hHSF1). (B) HSF1 protein expression by Western blot analysis. Multiple HSF1 bands were observed in retinal lysates since mouse HSF1 splice variants encode proteins of different lengths. (C) Representative images of retinal sections labeled with HSF1 antibody (green). Blue is DAPI staining. Scale bar: 50 μm.

To evaluate the role of HSF1 on retinal neuronal cell survival after ischemia, we introduced transgenic mice overexpressing the full-length human HSF1 gene (HSF1-Tg),^6^ which express HSF1 mRNA and protein 2- to 4-fold higher than wild-type (WT) littermates across a variety of tissues including the central nervous system.^6^ Since the retinal expression of HSF1 in this mouse strain is unknown, we first examined its expression at mRNA and protein levels. Quantification of mRNA for human, murine, and murine/human HSF1 by qPCR revealed that human HSF1 transcript was expressed in the retina of HSF-Tg mice while endogenous mouse HSF1 mRNA is not altered compared with WT mice (Fig. 2A). Total HSF1 mRNA (murine and human) was 2.5-fold higher in HSF-Tg mice than that in WT mice (Fig. 2A). Similarly, Western blot and immunostaining analyses further confirmed a higher level of HSF1 in the retina of HSF-Tg mice (Figs. 2B, 2C).

**Figure 3:**
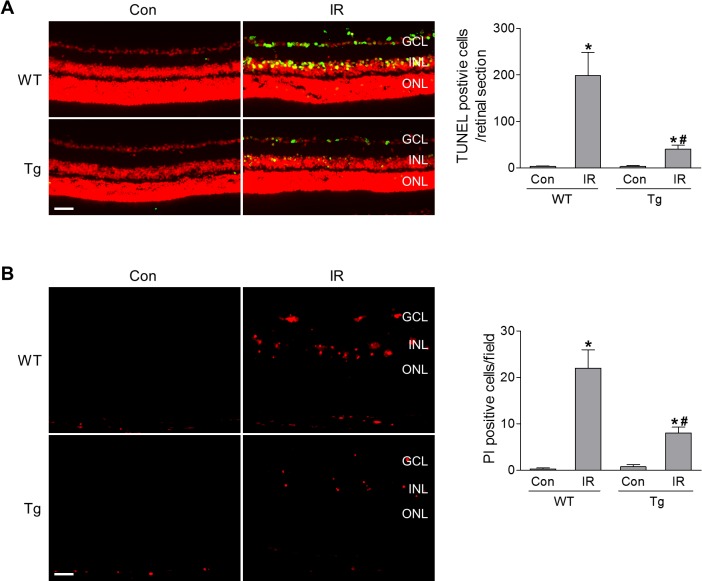
HSF1 overexpression reduces IR-induced both apoptotic and necroptotic cell death. WT and HSF1-Tg mice were subjected to IR. (A) TUNEL assay was conducted on retinal frozen sections at 12 hours after IR. Green: TUNEL-positive cells; red: PI staining of nuclei. Bar graph represents the number of apoptotic cells per retinal section. (B) Representative images of PI-labeled necroptotic cells (red) in retinal frozen section. PI was injected (IP) at 9 hours after IR, and eyeballs were collected at 3 hours after injection for retinal section. Bar graph represents the number of PI-positive cells per field. N = 4 to 5 mice; *P < 0.05 versus respective control, #P < 0.05 HSF1-Tg IR versus WT IR. Scale bar: 50 μm.

Next, we subjected HSF1-Tg mice and littermate WT mice into IR model. At 12 hours post-IR injury, we analyzed retinal cell apoptosis by TUNEL assay and found that TUNEL-positive staining was largely increased in neurons in the GCL and INL of WT retinas (Fig. 3A). This finding is consistent with reported data from our lab and others.^16^,^23^ Previous studies found that cell types undergoing IR-induced cell death include RGCs,^2^,^16^,^23^–^25^ amacrine cells,^24^,^26^ and bipolar cells.^27^ Notably, HSF1 overexpression dramatically decreased TUNEL staining after IR (Fig. 3A). In addition to apoptosis, increasing evidence has implicated the involvement of necroptosis in retinal cell death.^2^,^28^–^30^ Therefore we further assessed the effect of HSF1 overexpression on IR-induced necroptosis by PI uptake, in that PI only enters necrotic cells but is excluded by viable cells.^2^,^21^ We observed abundant PI-positive necrotic cells primarily in the GCL and INL of WT retinas at 12 hours after IR injury; these were markedly reduced in HSF1-Tg-IR retinas (Fig. 3B).

**Figure 4:**
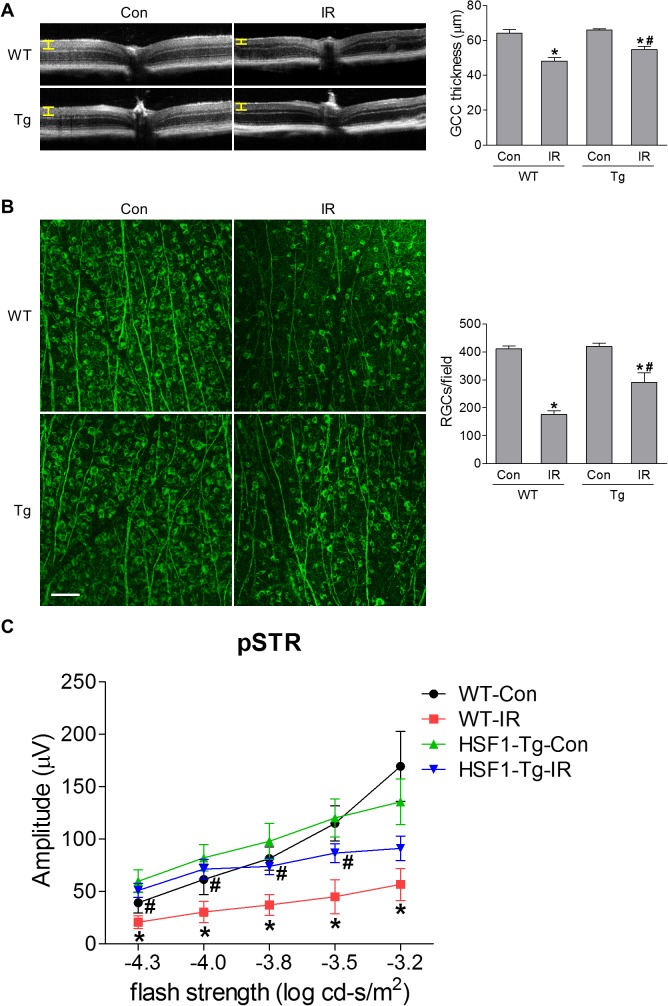
HSF1 overexpression prevents cellular/functional loss of RGCs after IR. WT and HSF1-Tg mice were subjected to IR. (A) OCT analysis in live mice for retinal thickness at 7 days after IR. Yellow H lines indicate the thickness of GCC (composed of the RNFL, GCL, and the inner plexiform layer [IPL]). Bar graph represents the thickness of GCC. (B) Representative images of retinal flatmounts labeled with Tuj1 antibody (green) at 7 days after IR. Scale bar: 50 μm. Bar graph represents the number of Tuj1-positive cells per field. (C) ERG analysis at 7 days after IR. Graph represents average amplitudes of pSTR over a range of stimulus strengths. N = 6 to 7 mice; *P < 0.05 versus respective control, #P < 0.05 HSF1-Tg IR versus WT IR. RNFL, retinal nerve fiber layer.

At 7 days after IR, the thickness of ganglion cell complex (GCC, including nerve fiber layer, GCL and inner plexiform layer), was measured by high resolution spectral domain optical coherence tomography (SD-OCT). It revealed that IR reduced the thickness of GCC compared to WT sham controls, indicating degeneration of RGCs and their axons. Overexpression of HSF1 blocked this decrease in retinal thickness after IR (Fig. 4A, Supplementary Fig. S2). To explore this observation further, we stained retinal flatmounts from WT and HSF1-Tg mice with Tuj1 antibody (a marker of RGCs), and imaged Tuj1-positive cells in the GCL by confocal microscopy. We found numbers of RGCs in the IR-exposed group were significantly decreased versus WT sham control not subjected to IR, while the RGCs in HSF1-Tg-IR group were preserved following IR-induced injury. These results suggest that HSF1 overexpression efficiently promoted RGC survival (Fig. 4B). To assess the function of the retina and in particular, the function of the remaining RGCs after IR, we employed ERG analysis and recorded the positive scotopic threshold response (pSTR) amplitudes at 7 days after IR. pSTR is generated at dim light and reflects RGC electrical function.^17^,^31^,^32^ pSTR values were comparable between WT and HSF1-Tg mice without IR. Significant decreases in amplitude were observed in the WT-IR retina at different stimuli compared with WT control. However, these decreases were substantially abrogated by HSF1 overexpression (Fig. 4C), indicating that the protection of RGCs with HSF1 overexpression also sustained RGC function. To test whether HSF1 was neuroprotective under other injury conditions in addition to IR, we examined RGC loss in a mouse TON model induced by optic nerve crush (ONC). Similarly, HSF1 overexpression significantly prevented ONC-induced RGC loss (Supplementary Fig. S3). These results implicate that HSF1 plays a protective role in injury-induced neurodegeneration in the retina potentially by regulating apoptosis and necroptosis.

### HSF1 Overexpression Boosts Hsp70 After IR Injury

**Figure 5:**
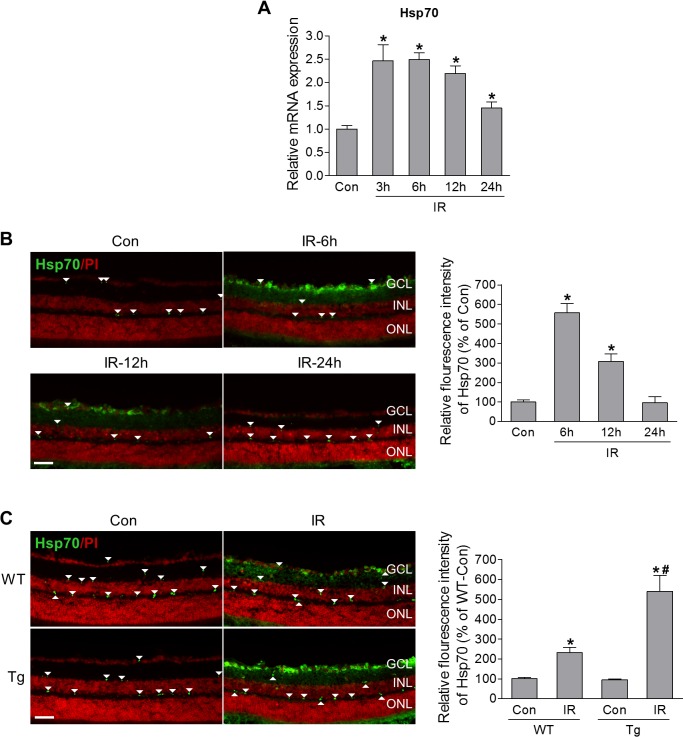
HSF1 overexpression enhances IR-induced Hsp70 expression. (A, B) WT mice were subjected to IR. At various time points after IR, retinas were collected for HSF1 mRNA expression by qPCR analysis (A) or eyeballs were collected for Hsp70 immunoreactivity (green) on retinal section (B). N = 3 to 5 mice; *P < 0.05 versus control. (C) Representative images of Hsp70 immunostaining (green) in retinal frozen sections. WT and HSF1-Tg mice were subjected to IR, and 12 hours later eyeballs were collected for retinal section. Red: PI staining. Arrowheads indicate nonspecific vessel staining. N = 3 to 4 mice; *P < 0.05 versus relevant control, #P < 0.05 HSF1-Tg IR versus WT IR. Scale bar: 50 μm.

HSF1 controls a distinct set of target genes in stressed cells, and Hsp70 is one of its major targets that protect retinal neurons.^9^ Therefore, we examined Hsp70 expression in the retina after IR injury. Real time PCR analysis revealed that upregulation of Hsp70 mRNA in the retinas occurred as early as 3 hours after IR injury, which was maintained at higher level at 6 and 12 hours after IR, followed by a decrease at 24 hours after injury (Fig. 5A). Similar to the pattern of HSF1 expression (Fig. 1B), the immunoreactivity of Hsp70 was increased in the GCL at 6 and 12 hours after injury, but returned to basal levels at 24 hours after injury (Fig. 5B). Therefore, similar to heat stress, IR injury can upregulate Hsp70 expression at both mRNA and protein levels in the retina. Moreover, before IR, even though HSF1 is overexpressed, it did not markedly affect Hsp70 protein levels compared to WT controls (Fig. 5C). However, after IR, overexpression of HSF1 further increased IR-induced Hsp70 expression (Fig. 5C), suggesting the HSF1 may regulate Hsp70 expression to exert its neuroprotective function in ischemic retinopathy.

### Heat Shock Response Relieves ER Stress After IR Injury

**Figure 6:**
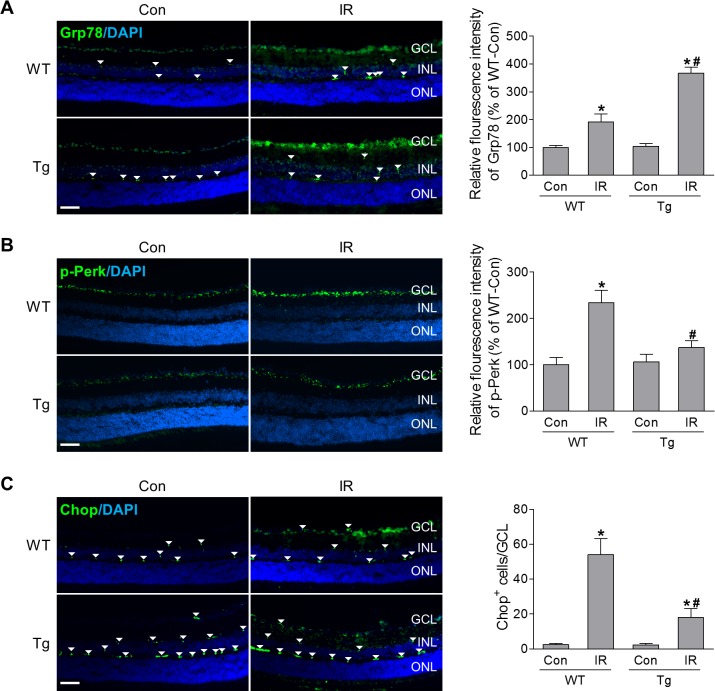
HSF1 overexpression alleviates IR injury-induced ER stress. Representative images of Grp78 (A), p-Perk (B) and Chop (C). Eyeballs were collected from WT and HSF1-Tg mice at 12 hours after IR and immunostaining was performed on retinal frozen sections. Arrowheads indicate nonspecific vessel staining. Bar graphs represent quantification of immunoreactivity of Grp78 and p-Perk, and the number of Chop-positive cells. N = 4 to 5 mice; *P < 0.05 versus relevant control, #P < 0.05 HSF1-Tg IR versus WT IR. Scale bar: 50 μm.

Both HSF1/HSPs and ER stress pathways participate in protein quality control. Studies from our laboratory and others have shown that ER stress play a role in IR-induced cell death.^16^,^18^ Overexpression of Grp78 (78-kDa glucose-regulated/binding immunoglobulin protein), which is a molecular chaperone located in the ER that regulates ER protein folding and translocation and controls activation of ER stress molecules, mitigates ER stress in RGCs during TON.^18^ Therefore, we evaluated whether HSF1 exerts its neuroprotective role against IR by regulating ER stress by examining the expression of ER stress-related molecules (Grp78, p-Perk, and Chop) in relation to HSF1/Hsp70. In comparison to WT sham control, Grp78 immunoreactivity was markedly increased and mainly localized in the neurons localized in the GCL and INL of WT retinas at 12 hours after IR (Fig. 6A). Similarly, the immunoreactivity of p-Perk and the number of Chop-positive cells in the GCL were also increased in WT-IR, indicating ER stress was induced in the retina after IR (Figs. 6B, 6C). However, compared with WT-IR, in IR-injured retinas with HSF1 overexpression, Grp78 immunoreactivity was further enhanced, which shares a similar pattern to the expression of Hsp70 (Fig. 5C), but p-Perk immunoreactivity and Chop-positive cells were markedly reduced compared to that of WT-IR retinas (Fig. 6). These data suggest that HSF1 may boost Grp78 expression to alleviate ER stress in ischemia retinopathy.

### HSF1 Overexpression Attenuates Tau Phosphorylation in the Retina After IR Injury

**Figure 7:**
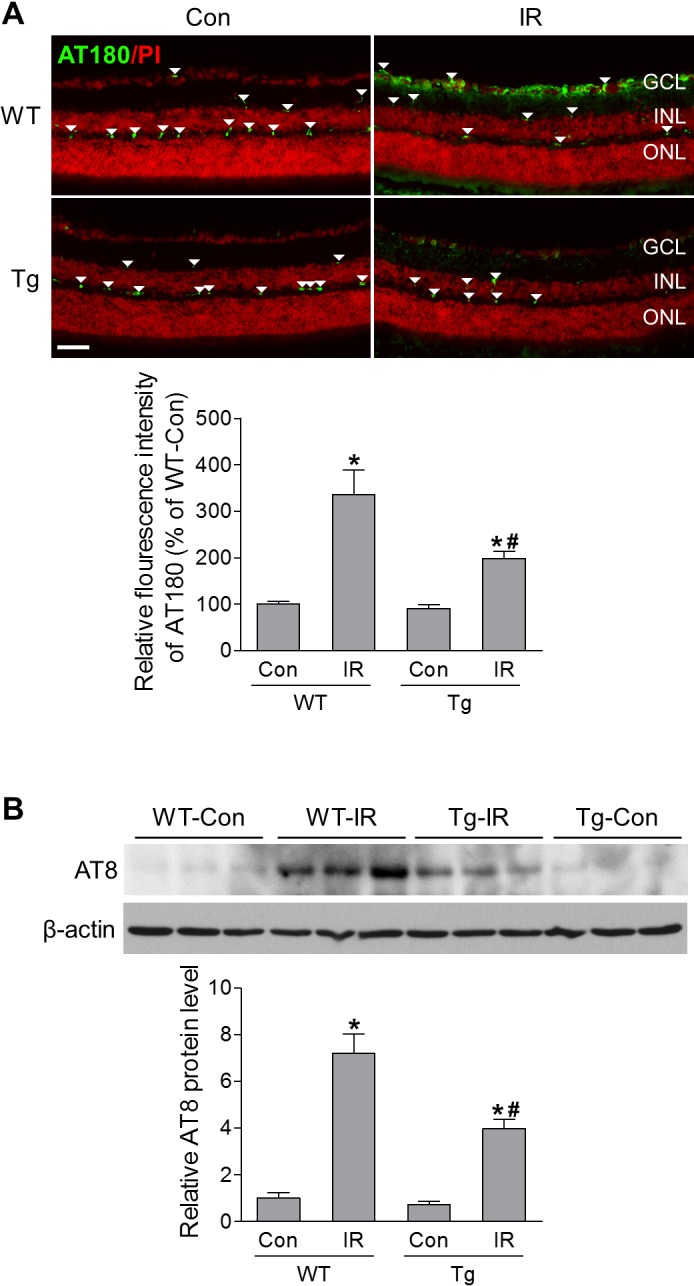
HSF1 overexpression abrogates IR-induced tau phosphorylation. WT and HSF-Tg mice were subjected to IR. Eyeballs for frozen sections or retinas for Western blot were collected at 12 hours after IR. (A) Representative images of phosphorylated tau at Thr231 (green) recognized by AT180. Arrowheads indicate nonspecific vessel staining. Bar graph represents quantification of immunoreactivity of phosphorylated tau in the retina. Scale bar: 50 μm. (B) Western blot analysis of phosphorylated tau at both serine 202 and threonine 205 recognized by AT8. N = 3 to 4 mice; *P < 0.05 versus relevant control, #P < 0.05 HSF1-Tg IR versus WT IR.

Both chaperone protein Hsp70 and ER chaperone Grp78 help ensure proper protein folding, including tau, which is a soluble microtubule-associated protein important for the maintenance and function of axons by binding to and stabilizing microtubules.^18^,^33^ Tau hyperphosphorylation and aggregation, defined as tauopathy, is a key player in many neurodegenerative diseases in the CNS.^33^ Recently we discovered that tauopathy also plays a critical role in RGC death and overexpressing Grp78 abolished tauopathy.^18^ Therefore, we investigated whether HSF1 overexpression can affect tau phosphorylation in IR. AT180 was used to examine tau phosphorylation at Thr231, which is critical for tau's hyperphosphorylation and aggregation. By immunostaining, we found a significant increase in tau phosphorylation in the GCL of WT mice at 12 hours after IR injury; however, it was remarkably reduced in the retinas of HSF1-Tg IR mice (Fig. 7A). Phosphorylated tau (Ser202/Thr205), as determined by Western blot with antibody AT8, was also increased in WT-IR retinas and abrogated by HSF1 overexpression (Fig. 7B). Taken together, these data suggest that HSF1overexpression may prevent RGC death through the modification of IR-induced tau phosphorylation.

### HSF1 Overexpression Attenuated Retinal Inflammation After IR Injury

**Figure 8:**
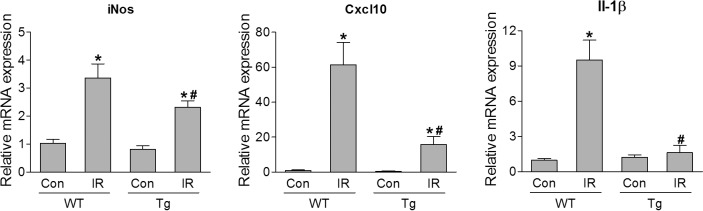
HSF1 overexpression relieves inflammation in the retina after IR injury. Quantitative PCR analysis for mRNA expression of inducible nitric oxide synthase (iNos), Cxcl10 and Il-1β. WT and HSF-Tg mice were subjected to IR, and retinas were collected at 12 hours after IR to extract RNA. N = 3 to 4 mice; *P < 0.05 versus relevant control, #P < 0.05 HSF1-Tg IR versus WT IR.

**Figure 9:**
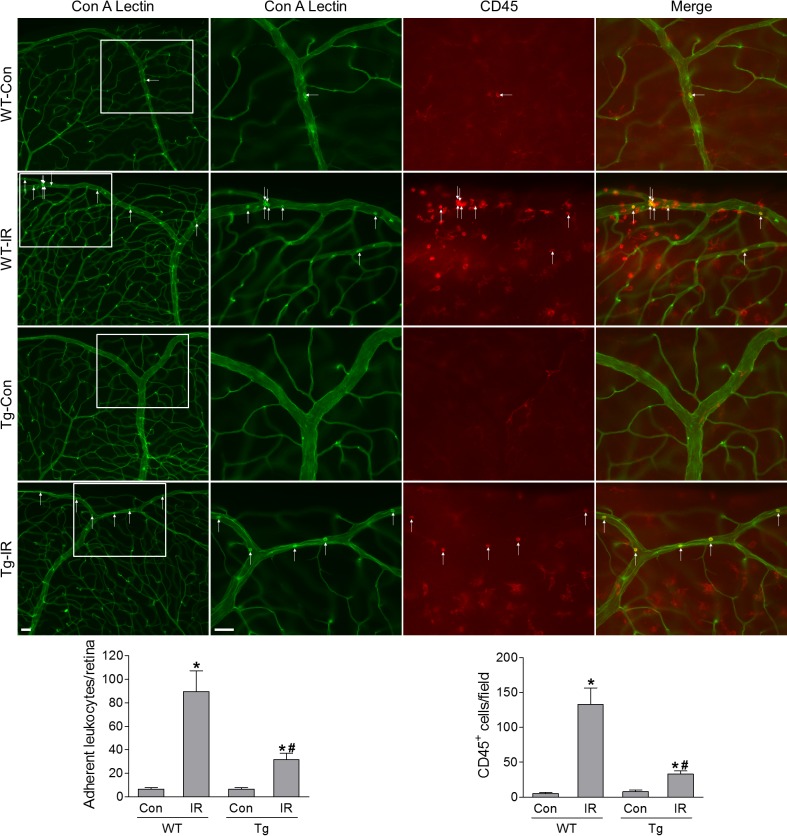
HSF1 overexpression decreases leukostasis in the retina after IR injury. Representative images of leukostasis in the peripheral retinas. WT and HSF1-Tg mice were subjected to IR and leukostasis assay was performed at 24 hours after IR. Green: Con A-labeled retinal vasculature and adherent leukocytes. Red: CD45 immunostaining for leukocytes. Rectangle in the left rows of images are zoomed in and arrows indicate stationary leukocytes adherent to the vascular endothelium. Bar graphs represents the number of leukocytes adherent to the retinal vasculature (Con A^+^, CD45^+^) per retina and infiltrated leukocytes (Con A^−^ , CD45^+^) per field. N = 4 mice; *P < 0.05 versus relevant control, #P < 0.05 HSF1-Tg IR versus WT IR.

A robust inflammatory response, signaled by a rapid rise in the levels of cytokines and chemokines, develops acutely following retinal IR, which also contributes to retinal neuronal injury.^16^ Therefore, we examined inflammatory cytokine and chemokine mRNA levels via qPCR, and found significant increases in iNos, Cxcl10, and Il-1β in WT retinas at 12 hours after IR (Fig 8). These changes were substantially abrogated by HSF1 overexpression (Fig. 8). Next, we utilized perfusion-labeling technique to assess the effects of HSF1 overexpression on leukostasis, a major component of inflammatory processes. At 24 hours after IR injury, retinal vasculature and leukocytes attached to vessels were perfusion-labeled with FITC-conjugated concanavalin A lectin (Con A). The identity of adherent leukocytes with Con A labeling was further confirmed by immunostaining with antibody against CD45, a marker for leukocytes (Fig. 9, Supplementary Fig. S4). We observed that all Con A–labeled static cells within retinal vessels were CD45^+^, indicating these cells are leukocytes. Adherent leukocytes (Con A^+^, CD45^+^ cells) in the whole retina and leukocytes infiltrated into the retina (Con A^−^, CD45^+^ cells) were separately counted. Their numbers significantly increased in WT mice at 24 hours after IR injury, but were clearly attenuated by HSF1 overexpression (Fig. 9). Together, these data indicate that HSF1 may exert its protective effects on retinal neurons against IR by inhibiting inflammatory response.

## Discussion

In this study, we found that HSF1 expression and activity were transiently upregulated after ischemia. Using a transgenic mouse that overexpresses the full-length human HSF1 gene (HSF1-Tg), we provide the first evidence that activation of HSF1 was an endogenous self-protective mechanism that prevents retinal neuronal injury after ischemic stress. Our data clearly demonstrated that overexpression of HSF1 activated Hsp70 expression, alleviated ER stress and tauopathy, prevented retinal inflammation, and protected retinal neuronal cells from death and dysfunction in a mouse model of retinal ischemia. Moreover, the neuroprotective effect of HSF1 was not limited to IR model since a similar protective effect was observed in a mouse model of optic nerve crush. This study highlights the potential value of boosting endogenous protective mechanisms including HSF1 activation for treating retinopathy. Although elevation of individual HSPs such as Hsp70, α-crystallins, Hsp40, and Hsp110 is neuroprotective in a variety of disease models,^8^–^13^ activation of HSF1 may be a more effective approach to save neurons than individual HSP since it induces the expression of a group of HSPs that work coordinately and synergistically to reestablish protein homeostasis and protect cells during stress. The function of HSF1 is modulated at multiple levels including regulation of its expression by noncoding RNAs, inhibition of its activation by binding to HSPs, and regulation of its transcriptional activity and protein stability by posttranslational modification (e.g., phosphorylation and acetylation).^5^ This complexity of regulation offers an opportunity to pharmacologically intervene in HSF1 activation at different levels. Ideally, a combination therapy that targets different mechanisms for HSF1 activation may strongly boost HSF1 activation while avoiding side effects arising from completely blocking or activating a single mechanism.

Because Hsp90 is one of the HSF1 client proteins that keep HSF1 in an inactive state, Hsp90 inhibitors were used to study the effects of HSF1 in vivo.^5^,^15^,^34^ Alternatively, other small compounds that have the ability to enhance HSF1 transcriptional activity or prevent its degradation were used.^5^,^35^–^37^ These approaches have provided valuable insights about the neuroprotective role of HSF1 in neurodegenerative diseases as well as in retinal degeneration.^5^,^15^,^34^–^37^ Nevertheless, a general limitation of chemical compounds is that they may have multiple downstream targets in addition to HSF1. For example, although Hsp90 inhibition with 17-AAG enhances visual function and delays photoreceptor degeneration in both P23H and R135L transgenic rat models of retinitis pigmentosa, the effect of 17-AAG on rod opsin P23H mutant is dependent on HSF1 but its effect on R135L mutant is mediated by a requirement of Hsp90 for rhodopsin kinase (GRK1) maturation and function.^15^ To overcome the limitation of using chemical compounds, we used a HSF1 transgenic mouse strain that overexpress the human full-length non-mutant HSF1 gene. While mice overexpressing a truncated constitutively active HSF1 under the control of a human β-actin promoter fail to overexpress HSF1 in the CNS and the male transgenic mice are infertile,^38^,^39^ our HSF1-Tg mice grow and breed normally as WT mice, and exhibit moderate increase in HSF1 mRNA and protein expression in all tissues including the CNS^6^ and the retina (Fig. 2). These mice have been shown to be protective from neurodegeneration in mouse models of Alzheimer's disease and amyotrophic lateral sclerosis.^40^,^41^ Using these mice, we provide direct evidence that HSF1 is a neuroprotective molecule after retinal ischemic injury. Moreover, human HSF1 transgene-mediated induction of Hsp70 protein is under the control of ischemic stress. These results suggest the HSF1-Tg mice is a very useful tool when assessing the pathophysiological roles of HSF1 in vivo.

**Figure 10:**
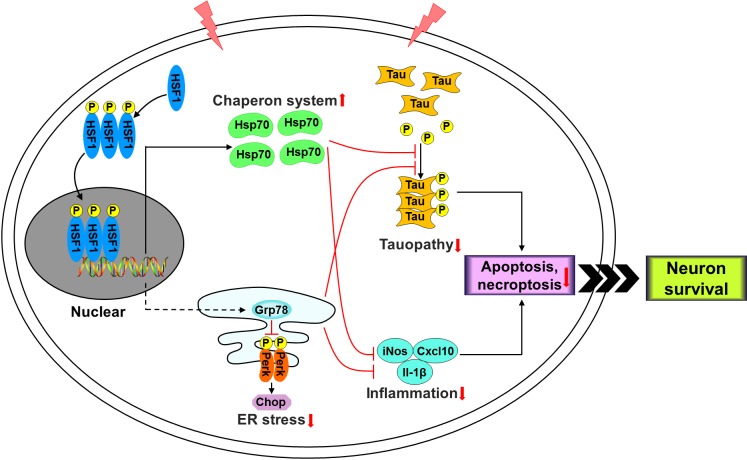
Schematic representation of the HSF1 pathway in retinal neurons after IR.

Although mechanisms by which HSF1 protects RGCs after retinal ischemia remain to be further investigated, our current study suggests that multiple mechanisms are involved in this process (Fig. 10). HSPs are critical components of a complex defense mechanism that promotes cell survival during stress.^9^ HSPs in particular Hsp70 execute cell protective role by maintaining protein homeostasis through assisting cytosolic protein folding, assembly, translocation, repair and degradation or by inhibiting apoptotic and necrotic pathways.^9^ Boosting Hsp70 level has been shown to reduce brain injury after global and focal ischemia, and prevent RGC death induced by increased intraocular pressure and optic nerve crush.^9^ Since HSF1 is the master transcription factor that controls expression of HSPs, it is possible that the neuroprotective effect of HSF1 is directly mediated by HSPs, as is demonstrated by our data that HSF1 overexpression markedly enhanced Hsp70 expression after retinal ischemia but did not induce its expression under normal condition. Other than HSPs, HSF1 may protect RGCs by alleviation of ER stress which is another mechanism for protein homeostasis in response to misfolded proteins accumulated in the ER but induces cell death and tissue injury when it is severe or prolonged.^16^,^18^ We found that HSF1 overexpression repressed IR-induced Perk phosphorylation and Chop expression, indicating HSF1 represses ER stress pathways that lead to cell death.^18^ Mechanistically, these effects may involve upregulation of Grp78, an ER chaperone protein repressing ER stress pathways and protecting RGCs during stress,^18^ by HSF1 overexpression. To our knowledge, this is the first observation that a molecule simultaneously regulates two pathways involved in protein homeostasis in ischemic retinopathy and therefore protects RGCs from injury. Our data are in line with what was found in yeast that there is interaction between HSF1 and ER stress pathways and constitutively active HSF1 relieves ER stress by facilitating ER translocation of newly synthesized polypeptides, enhancing the ERAD pathway, and promoting vesicular transport out of the ER.^42^

Tauopathy plays a critical role in AD and many other neurodegenerative disorders in the CNS, such as progressive supranuclear palsy and traumatic injury.^43^–^46^ We and others have recently demonstrated that tauopathy is a key player in RGC injury in models of TON, glaucoma and AD.^18^,^47^–^49^ Moreover, overexpressing Grp78 not only blocked tauopathy but also saved RGCs after TON.^18^ Additionally, increased levels of Hsp70 and Hsp90 were shown to reduce tauopathy.^50^ These studies, together with our data showing overexpression of HSF1 reduced tau phosphorylation, suggest a model that HSF1 induces HSPs and Grp78 which attenuates RGC injury partially through blockade of tauopathy (Fig. 10). In addition to regulation of tauopathy, we found that HSF1 overexpression attenuated production of inflammatory molecules such as iNos, Cxcl10, and Il-1β and prevented leukocyte attachment and infiltration, which is a hallmark of vascular inflammation. Although inflammation is a necessary step for body to fight against pathogens and heal wounds, uncontrolled or excessive inflammation causes tissue injury and leads to diseases. Previous studies have shown that ER stress-regulated Cxcl10 production in retinal neurons contributes to retinal inflammation and neuronal injury after ischemic injury^16^ and intracellular HSPs exhibit anti-inflammatory properties.^51^ At present, it is unclear whether reduced retinal inflammation is directly caused by HSF1-mediated blockade of ER stress and upregulation of HSPs, or results from HSF1 working on other mechanisms that lead to an overall lower level of ischemic injury, and secondary to that, the cytokine levels and leukostasis differ significantly from WT. If it is the direct effect of HSF1 overexpression, our study would suggest a model that HSF1 induces HSPs, inhibits ER stress, prevents inflammation and therefore protects retinal neurons (Fig. 10).

In summary, our data reveal that upregulation of HSF1 is a self-protecting mechanism. The pleiotropic role of HSF1 warrants further investigation of whether it exhibits similar neuroprotective functions in other models of retinal diseases such as diabetic retinopathy, glaucoma, retinopathy of prematurity, and retinitis pigmentosa. Considering that numerous small molecules that can activate HSF1 are available,^52^ pharmacologically boosting HSF1 activity could be beneficial in a variety of retinal diseases.

## Supplementary Material

Supplement 1
