# Supplementary material for: Neuroprotective Effects of HSF1 in Retinal Ischemia-Reperfusion Injury
Source: Invest Ophthalmol Vis Sci. 2019 Mar;60(4):965–77. doi: 10.1167/iovs.18-26216 (PMC6424471; doi:10.1167/iovs.18-26216)
Supplement: Supplement 1 [file iovs-60-04-03_s01.pdf]

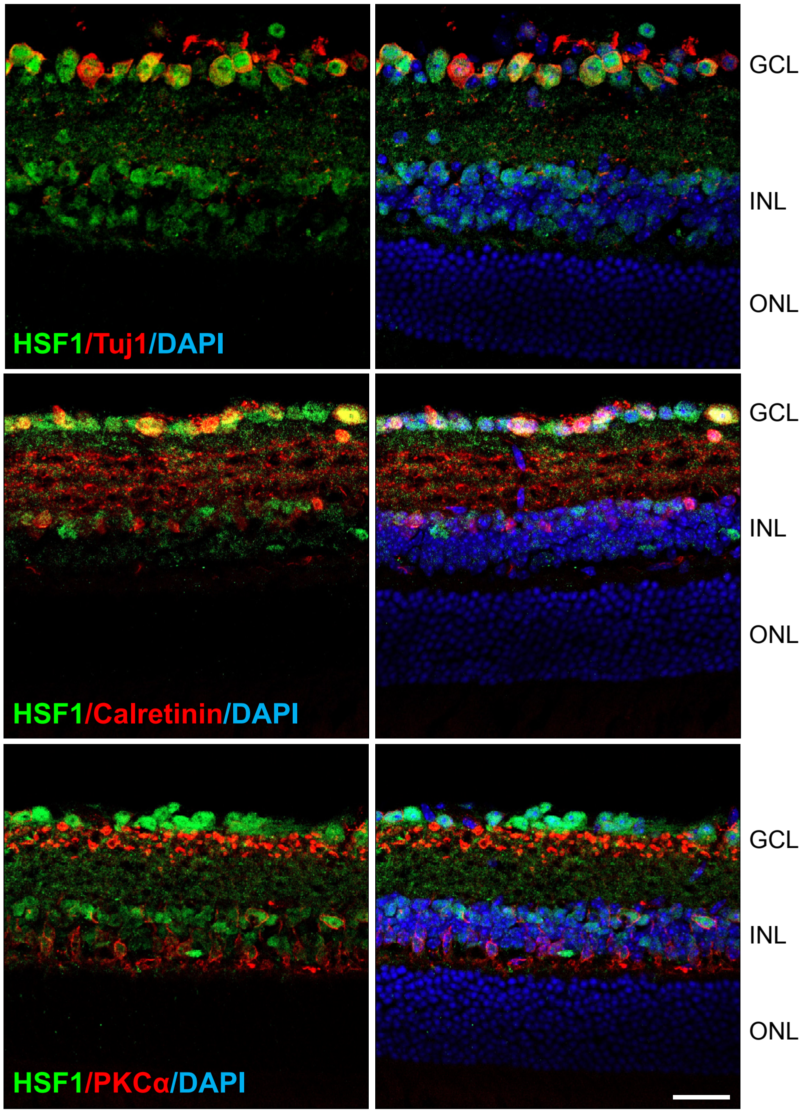

Supplementary Fig. S1. HSF1 expression in the retina. Retinal sections from WT mice at 6 hours after IR were stained with antibodies against HSF1(green) and different retinal cell markers (red): Tuj1, RGC maker; Calretinin, amacrine cell marker; PKCα, bipolar cell marker. Blue: DAPI staining for the nuclei. Scale bar, 25 μm. GCL, ganglion cell layer; INL, inner nuclear layer; ONL, outer nuclear layer.

Supplementary Fig. S1

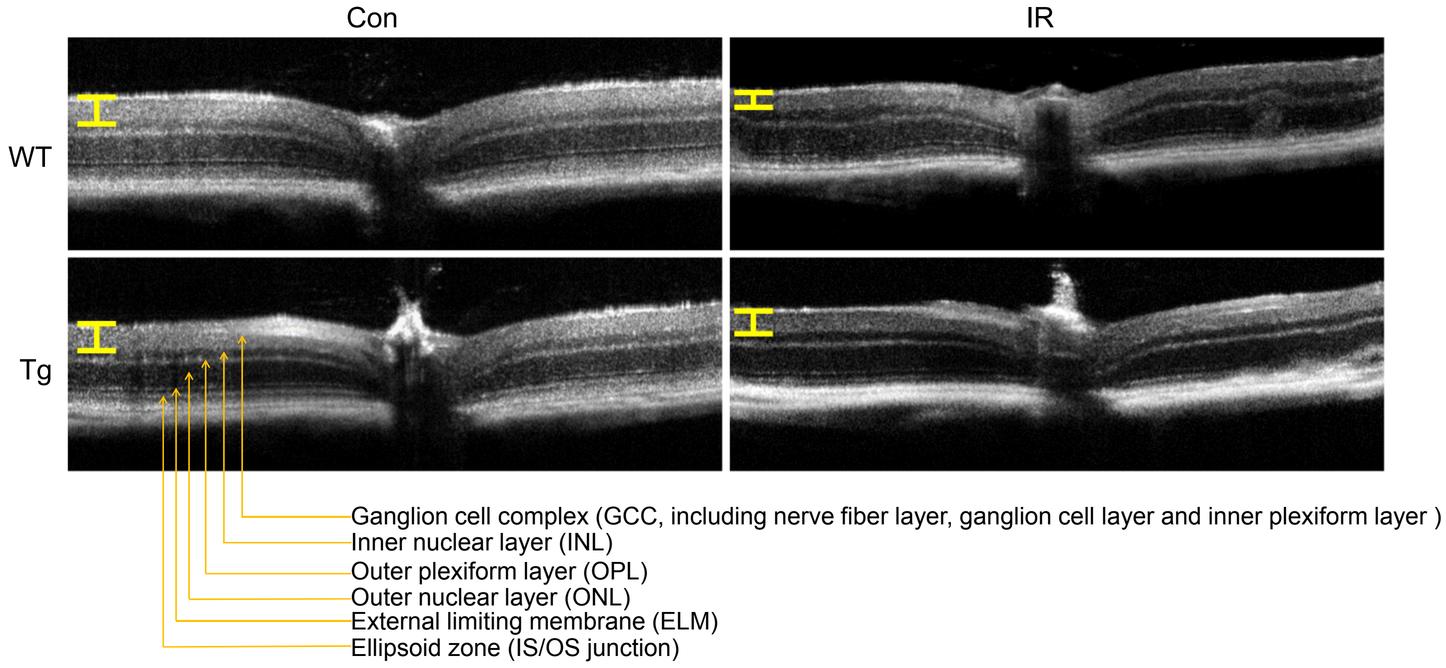

Supplementary Fig. S2. Enlarged OCT images of Figure 4A .

Supplementary Fig. S2

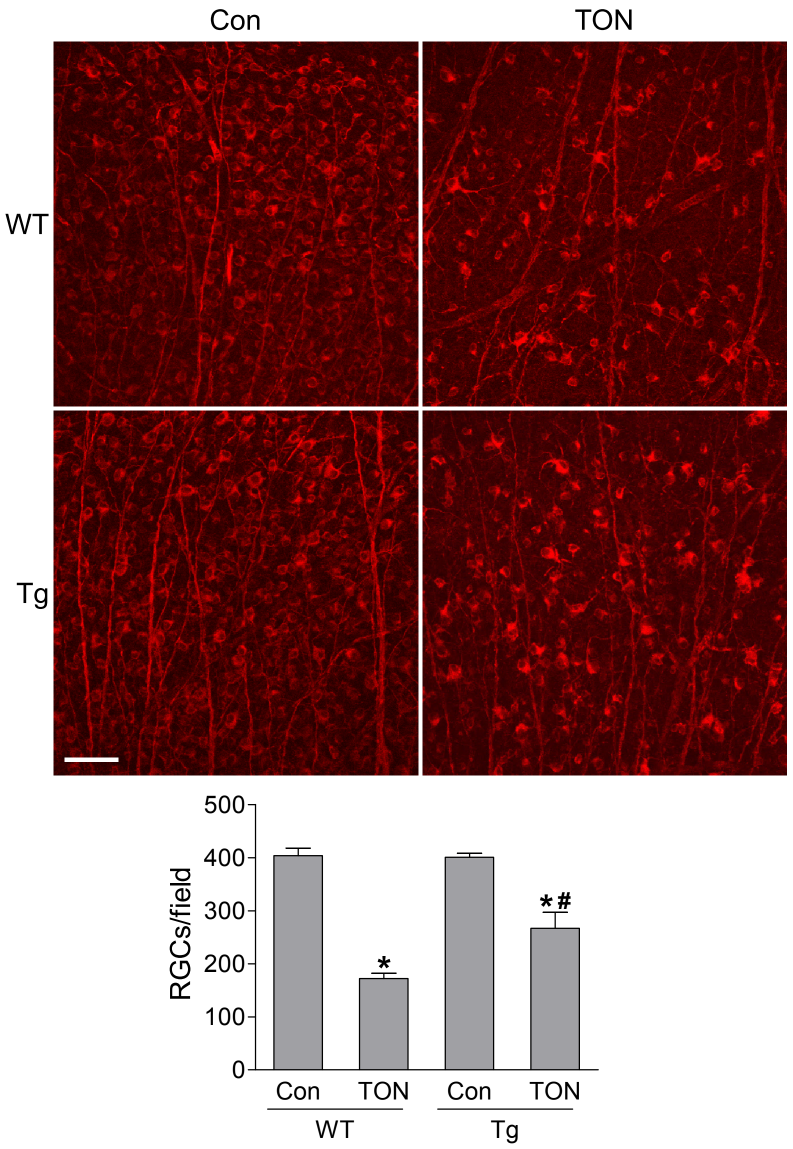

Supplementary Fig. S3. HSF1 overexpression prevents TON-induced loss of retinal ganglion cells. Representative images of retinal flatmounts labeled with Tuj1 antibody (red) at 7 days after TON. Bar graph represents the number of Tuj1-positive cells per field. N=3-4 mice; *P<0.05 versus respective control, #P<0.05 HSF1-Tg-TON versus WT-TON. Scale bar: 50 μm.

Supplementary Fig. S3

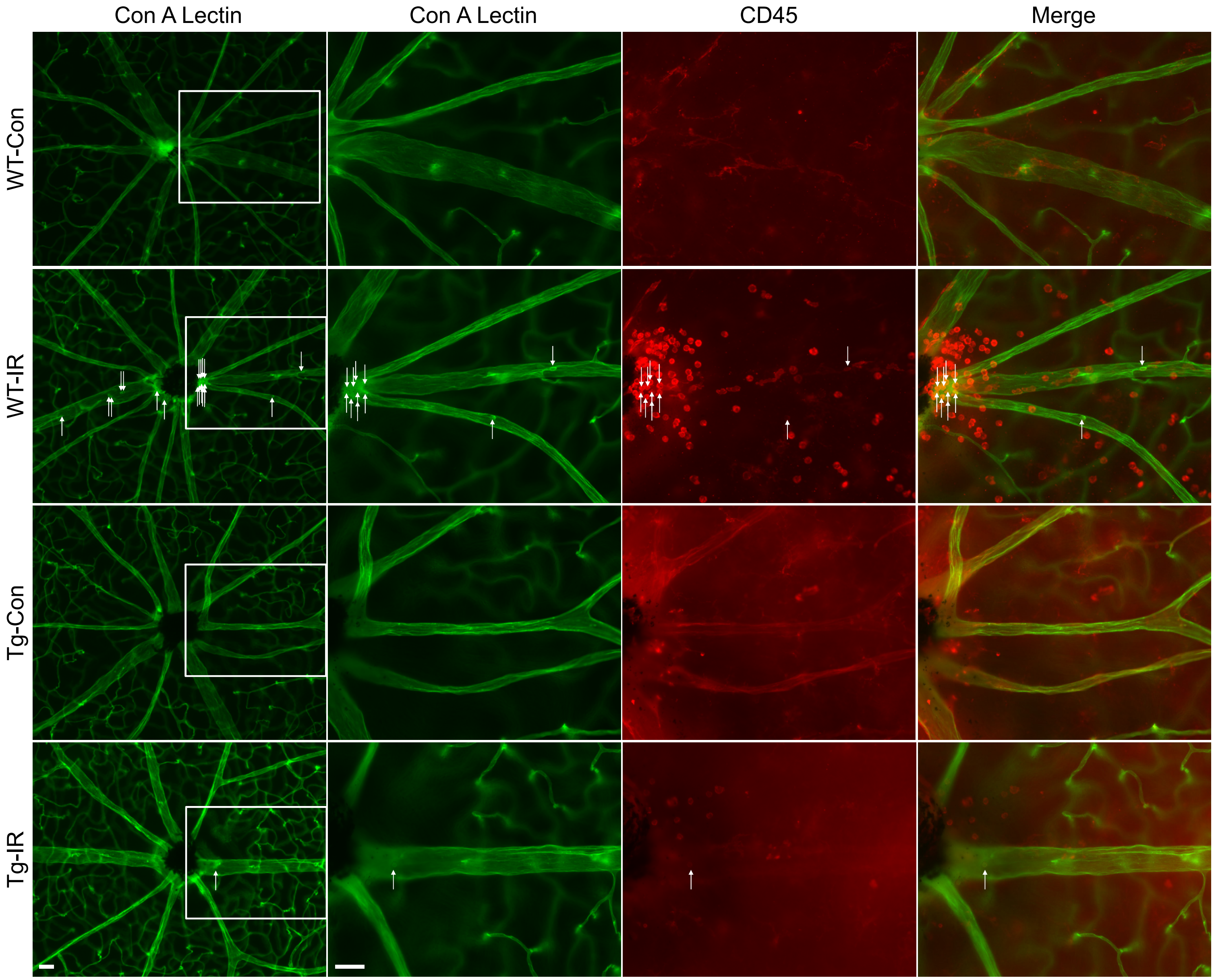

Supplementary Fig. S4. HSF1 overexpression decreases leukostasis in the retina after IR injury. Representative images of leukostasis in the central retinas. WT and HSF-Tg mice were subjected to IR and leukostasis assay was performed at 24 hours after IR. Green: Con A-labeled retinal vasculature and adherent leukocytes. Red: CD45 immunostaining for leukocytes. Rectangle in the left rows of images are zoomed in and arrows indicate stationary leukocytes adherent to the vascular endothelium.

Supplementary Fig. S4
